# Distinct spatiotemporal patterns between fungal alpha and beta diversity of soil–plant continuum in rubber tree

**DOI:** 10.1128/spectrum.02097-24

**Published:** 2024-12-27

**Authors:** Yaqing Wei, Zhixiang Wu, Guoyu Lan

**Affiliations:** 1 Rubber Research Institute, Chinese Academy of Tropical Agricultural Sciences, Haikou, Hainan, China; 2 Hainan Danzhou Tropical Agro-Ecosystem National Observation and Research Station, Danzhou, Hainan, China; Ruhr-Universitat Bochum, Bochum, Germany

**Keywords:** rubber tree, fungi, diversity, spatiotemporal patterns

## Abstract

Plant-associated microbial communities strongly relate to host health and productivity. Still, our knowledge of microbial community spatiotemporal patterns in soil–plant continuum is largely limited. Here, we explored the spatiotemporal dynamics of fungal communities across multiple compartments (phyllosphere, leaf endosphere, soil, rhizosphere, rhizoplane, and root endosphere) of rubber tree in two contrasting seasons collected from Hainan Island and Xishuangbanna. Our results demonstrate that the fungal alpha and beta diversity exhibited distinct pattern; the alpha diversity is highly dependent on seasonal changes, while beta diversity only showed a geographical variation pattern. The season-specific environmental factors (e.g., climatic factors) were the most important factors in shaping fungal alpha diversity across the soil–plant continuum. Physicochemical properties explained some of the microbial beta diversity spatiotemporal variation observed, with leaf phosphorus (P) and soil available potassium (AK) likely being the main factors that drove the geographical variation. We further identified the variation of edaphic (e.g., AK) and leaf physicochemical factors (e.g., P) were mainly caused by regional sites (*P* < 0.05). Taken together, our study provides an empirical evidence that the distinct spatiotemporal patterns of alpha and beta diversity of rubber tree fungal diversity and significantly expand our understanding of ecological drivers of plant-associated microbial communities.

**IMPORTANCE:**

Plants harbor diverse microorganisms in both belowground and aboveground compartments, which play a vital role in plant nitrogen supply and growth promotion. Understanding the spatiotemporal patterns of microbial communities is a prerequisite for harnessing them to promote plant growth. In this study, we show that the alpha and beta diversity of soil–plant continuum in rubber tree exhibited distinct spatiotemporal pattern. Alpha diversity is highly dependent on seasonal changes, while beta diversity only showed a geographical variation pattern. Climatic factors were the most important factors in shaping fungal alpha diversity. Leaf phosphorus (P) and soil available potassium (AK) were major drivers to induce geographical variation.

## INTRODUCTION

Microbial organisms inhabit all biomes of the Earth (1), and provide a number of life-support functions for their host (2). Therefore, we must develop a better understanding of the distribution and ecological drivers of aboveground and belowground microbial communities. Recent studies have demonstrated the immense role of plant compartments and environmental factors in driving the assembly of microbiomes (3–5). Geographic location and seasonal change have been demonstrated to influence community composition. For example, it was suggested that the assembly of the phyllosphere bacterial and fungal communities is predominantly determined by host compartment (epiphytic and endophytic) and site location (3). As for soil and rhizosphere, microbiomes are influenced by environmental factors (e.g., site, soil properties, and climate) (5–10). However, these studies mainly focused on a single niche or compartment, and a significant knowledge gap exists on how spatial heterogeneity versus time shapes the diversity and structure of microbial communities along the soil–plant continuum. Different scales have varying impacts on plant microorganisms (11–14). Moreover, these examples have shown that soil microbial communities are influenced by spatial or temporal change, but understanding of how seasonal changes (e.g., dry and rainy seasons) affect the compositions and diversity of soil–plant continuum microbial communities at the regional scale is still limited.

Rubber plantation is the most economically important agro-ecosystem in tropical China, particularly in Hainan Island and Xishuanbanna (abbreviated as Banna below) (15), which accounts for more than 90% of the total rubber plantation area of China (16). It is reported rubber plantations have multiplied quickly throughout Southeast Asia over the last two decades (17, 18). As far as we know, rubber forests account for almost 25% and 40% of the total vegetation area in Hainan Island and Banna (19), respectively. Previous work in Hainan has shown that seasonal change and site location were the dominant factors resulting in shifts in soil microbial composition at the local and geographic scales, respectively (6, 19–21). However, these studies were limited, particularly in the sample size scales used. Given the importance of microbes in functional roles in tropical forest ecosystem, such as nutrient acquisition, disease resistance, and stress tolerance (22), and the central part of rubber plantation of terrestrial ecosystems both in Hainan and Banna. Moreover, little attention has been paid to the plant-associated microbial communities on the same scale. As a result, it is difficult to describe the overall pattern of the soil–plant continuum. Therefore, it is necessary to study the spatiotemporal pattern and ecological drivers of the rubber tree soil–plant continuum microbial communities of these two locations.

In this study, we examine fungal communities across multiple compartments (bulk soils, rhizosphere, rhizoplane, root endosphere, phylloplane, and leaf endosphere) based on field samples of rubber trees during both dry and rainy seasons in two major areas of China (Fig. S1). We aimed to (i) determine the distribution pattern of fungal communities along the soil–plant continuum of rubber tree and (ii) identify the relative importance of spatial heterogeneity versus season and dominant drivers for driving fungal community at the regional scale. We hypothesized that (i) both geographic and seasonal factors will influence the assembly of rubber-associated fungal communities and (ii) the spatiotemporal distribution pattern is mediated by the spatiotemporal variation of the driving factors.

## MATERIALS AND METHODS

### Study site and sampling

The study was conducted in two locations: Hainan Island and Banna. Hainan Island experiences a tropical maritime monsoon climate, characterized by a rainy season from May to October and a dry season from November to April. Rubber plantations in Hainan are located at low latitudes and altitudes, making them tropical island-type plantations. On the other hand, Banna has a warm and humid climate throughout the year, with dry (November to April) and rainy seasons (May to October) similar to Hainan. The rubber plantations in Banna are situated at higher latitudes and altitudes, representing a tropical inland static wind plantation area type with fertile soil (19).

We selected six major plantation sites from Danzhou (DZ), Wanning (WN), and Ledong (LD) Districts in Hainan Island, as well as from Jinghong (JH), Menglun (ML), and Mengpeng (MP) Districts in Xishuangbanna (Fig. S1). At each site, we chose three plots separated by distances of 5–15 km for sampling, resulting in a total of 18 plots. The sample collections were carried out from 24 August to 24 September 2019 (rainy season) and 24 November to 23 December 2020 (dry season). Therefore, we obtained 36 soil samples from each plot, resulting in a total of 216 samples, including those from the phyllosphere, leaf endosphere, soil, rhizosphere, rhizoplane, and root endosphere compartments. For each leaf sample, we analyzed water content (WC), pH, leaf phosphorus (P), potassium (K), nitrogen (N), and organic matter (LOM). Additionally, we quantified soil total nitrogen (TN), total phosphorus (TP), total potassium (TK), organic matter (SOM), available potassium (AK), ammonium nitrogen (AN), nitrate nitrogen (NN), and available phosphorus (AP). The detailed methodology of the soil, root, and leaf sampling approach, as well as the analysis of physicochemical properties, are described in Method S1. We collected latitude, longitude, and elevation data for each site. Additionally, data on average monthly precipitation and average monthly temperature for each month were obtained from the National Meteorological Information Center (https://www.data.cma.cn).

### DNA extraction and sequencing

Microbial community DNA was extracted from soil, root, and leaf samples using the FastDNA Spin Kit for Soil (MP Biomedicals), following the manufacturer’s instructions. The hypervariable region ITS of the fungal gene was amplified using the primer pairs ITS1F (5′-CTTGGTCATTTAGAGGAAGTAA-3′) and ITS2R (5′-GCTGCGTTCTTCATCGATGC-3′) (23). Sequencing was conducted on the Illumina MiSeq platform, following standard protocols. All samples were pooled in equimolar concentrations and subjected to paired-end sequencing on the Illumina MiSeq platform. The paired-end sequences were merged to generate single sequences of approximately 300 bp. These sequences were then quality-filtered (maximum expected error = 0.2), and singletons were removed using USEARCH v.10 (24). Raw reads and operational taxonomic units (OTUs) obtained from the sequencing were deposited into the NCBI Sequence Read Archive (SRA) database (accession number: SRP342019) and Table S1.

### Data analysis

The raw fastq files were demultiplexed and subjected to quality filtering using QIIME (25) (version 1.17). OTUs were clustered at a 97% similarity cutoff using UPARSE version 7.1 (26). Chimeric sequences were identified and removed using UCHIME. The taxonomy of each representative sequence of the OTUs was determined using the RDP Classifier version 2.2 (27) against the ITS database with a confidence threshold of 0.7 (28).

Diversity indices were based on resampled sequences using the MOTHUR program (29). Alpha diversity (observed OTU richness, Shannon, and Evenness) index was calculated for each sample using the *vegan* package (30) in the R environment (version 4.3.1). We modeled the alpha diversity as a response variable and site location and seasonal change as fixed effects. The relative importance of the location and season for alpha diversity was evaluated by analysis of variance (ANOVA) (31), with the *P* values corrected using the false discovery rate method. Principal coordinate analysis was conducted using Bray-Curtis dissimilarity for the 216 samples to explore fungal community compositional differences (beta diversity) in different compartments, seasons, and locations, and then was visualized by using *ggplot2* (32). The relative importance of the locations/sites and two seasons for explaining the variation in environmental variables and the alpha diversity of fungi were evaluated by two-way ANOVA. The permutational multivariate analysis of variance in the R vegan package was used to test the variations in fungal beta diversity as explained by locations and seasons (33).

Random forest (RF) analysis (rfPermute function in *rfPermute* package in R) was used to identify the main environmental drivers for soil fungal alpha diversity (34, 35). To reveal the relationship between alpha diversity and environmental factors, a linear (linear least-squares regression analysis) or nonlinear regression was used based on RF results. The Mantel test was performed to evaluate the influence of soil properties, climate factors, and leaf properties upon fungal communities of different compartments, using the mantel function of the *ecodist* package (36) and *vegan* package for R and visualized by using the *linkET* package. We used variation partitioning analysis (VPA) to quantify the relative importance of seasonal change (dry and rainy season), climatic factors (temperature and precipitation), physicochemical properties (soil including soil pH, WC, SOM, TK, TN, TP, AN, NN, AK, and AP; leaf including pH, WC, LOM, K, N, and P) and geographic variables (37). Latitudinal and longitudinal data for each site was transferred to rectangular data to represent spatial distance by function pcnm of *Vegan* package, and VPAs were conducted with function varpart in the *vegan* package for R (30).

## RESULTS

### Community composition and environmental variables

A total of 9,036, 7,861, 11,001, 10,261, 9,701, and 3,846 OTUs were detected for the six compartments (phyllosphere, leaf endosphere, soil, rhizosphere, rhizoplane, and root endosphere, respectively). Among these OTUs, the majority were assigned to the classes Dothideomycetes (18.10%), Sordariomycetes (16.51%), Eurotiomycetes (10.64%), Tremellomycetes (9.46%), and Agaricomycetes (8.37%) at the class level (Fig. S3).

**Fig 1:**
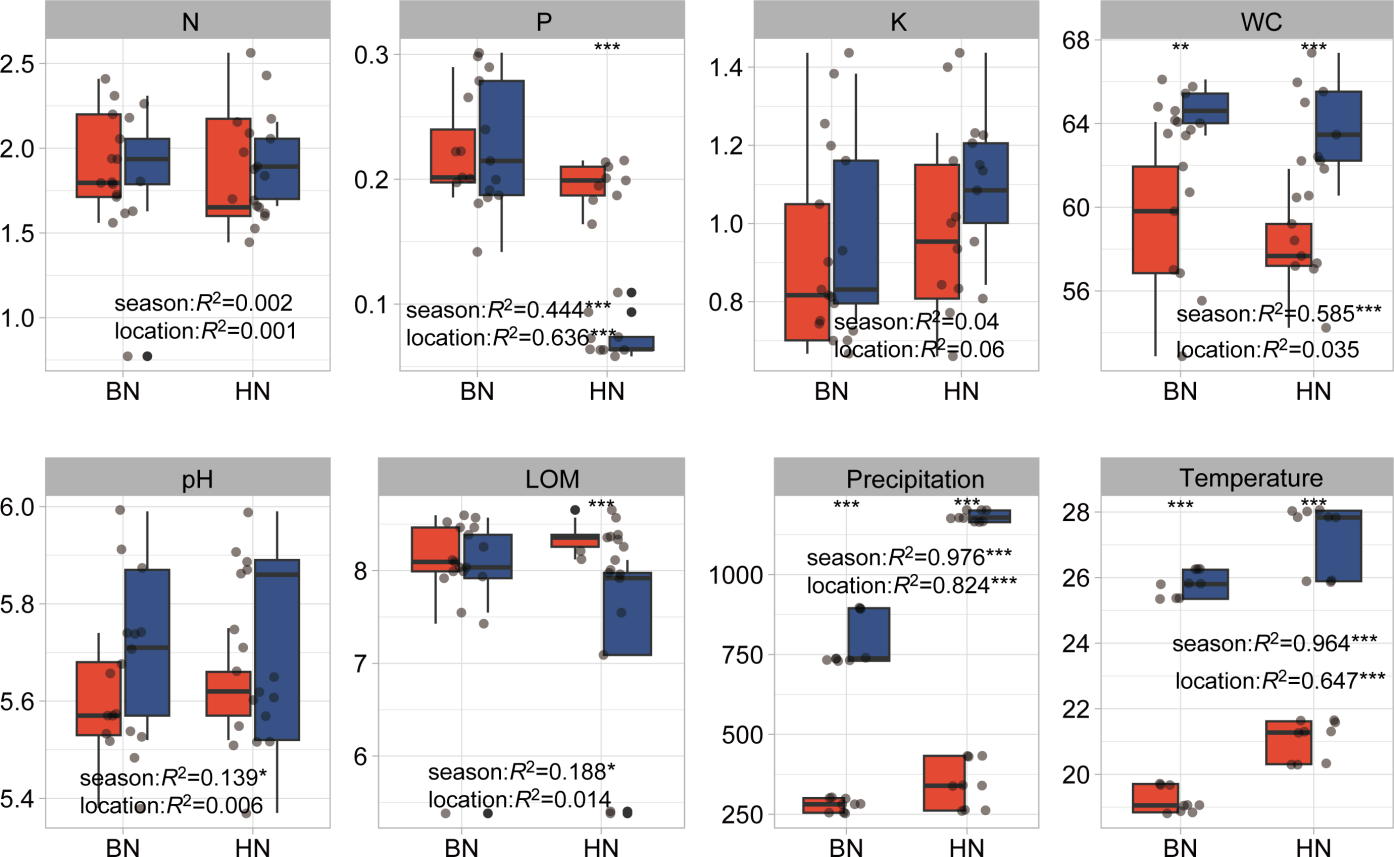
Environmental variables in dry and rainy seasons between two locations of rubber tree leaves. The significant differences between seasons and locations were detected by two-way ANOVA. K, total potassium; LOM, leaf organic matter; N, total nitrogen; P, total phosphorus; WC, water content. *, *P* < 0.05; **, *P* < 0.01, ***, *P* < 0.001.

**Fig 2:**
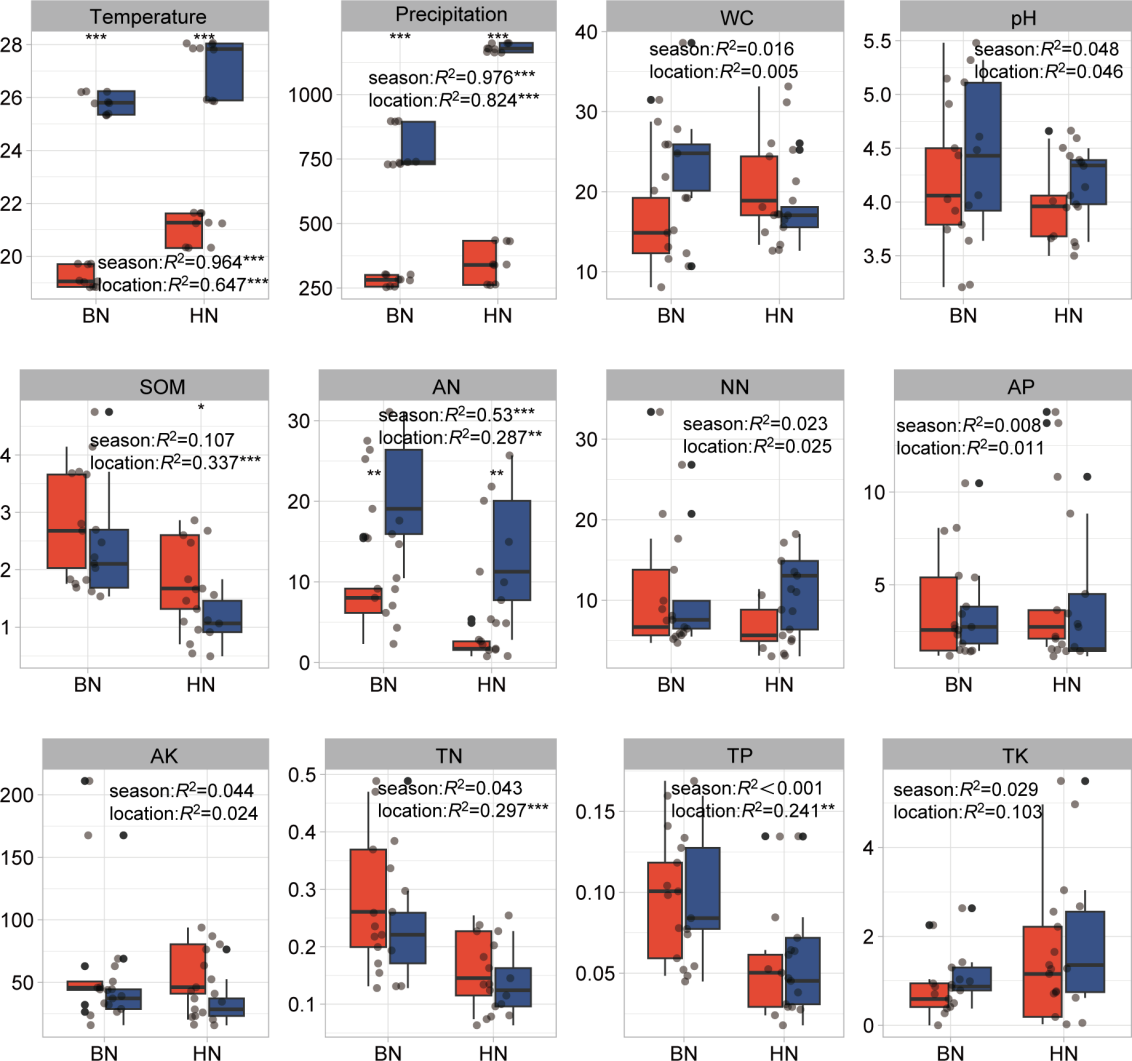
Environmental variables in dry and rainy seasons between two locations of rubber tree soils. The significant differences between seasons and locations were detected by two-way ANOVA. AK, available potassium; AN, ammonium nitrogen; AP, available phosphorus; NN, nitrate nitrogen; pH, Soil pH; SOM, soil organic matter; TK, total potassium; TN, total nitrogen; TP, total phosphorus; WC, water content. *, *P* < 0.05; **, *P* < 0.01, and ***, *P* < 0.001.

Among the six measured leaf environmental variables, WC, leaf pH, and organic matter (LOM) showed strong seasonal variations, with higher values during the rainy season compared to the dry season. Leaf phosphorus (P) variables, on the other hand, were more influenced by geographical location or site rather than seasonal changes (Fig. 1; Fig. S3). While there were significant seasonal changes of N in LD, ML, and K in DZ, the ANOVA analysis was not found to be significantly affected by the seasons (Fig. S3). In contrast, the 10 measured soil physicochemical properties were primarily explained by geographical locations or sites. Variables, such as AK, soil pH, TN, TK, TP, WC), SOM, NN, and AP, were largely influenced by the specific sites where sampling was conducted. In addition, mean seasonal precipitation and mean seasonal temperature showed significant seasonal variations. Approximately 97.6% (*P* < 0.001) of the variation in precipitation and 96.4% (*P* < 0.001) of the variation in temperature were explained by sampling seasons. During the rainy season, leaf WC, precipitation, and temperature were significantly higher compared to the dry season (Fig. 2; Fig. S4). In the rainy season, temperature, precipitation, and AN were significantly higher than the dry season in all sites. And WC in JH, pH in WN, were significantly higher, yet WC in DZ, SOM in LD, and TN in MP were significantly lower than the dry season (Fig. S4).

### Spatiotemporal pattern of the fungal community

**Fig 3:**
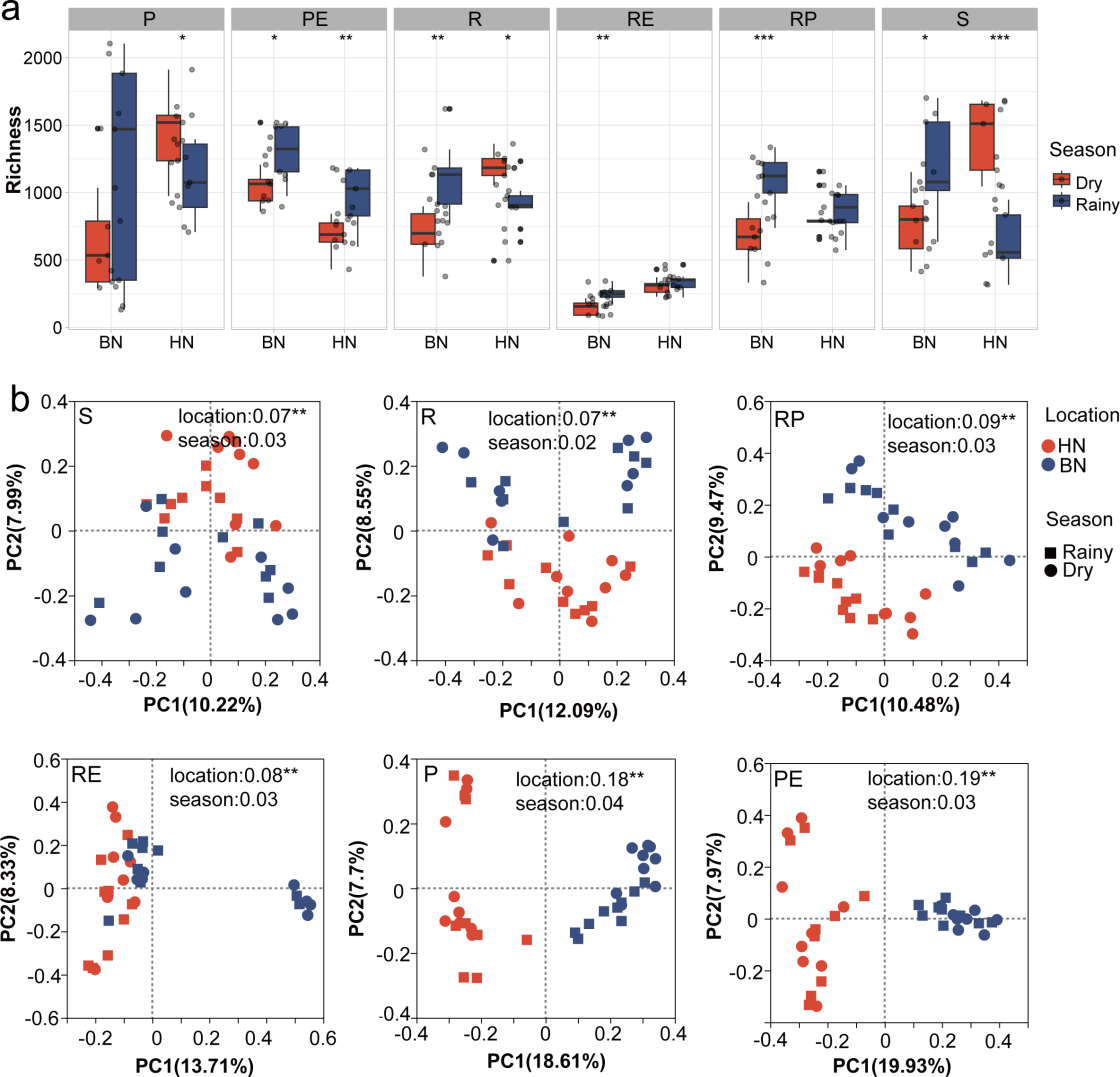
Alpha diversity of rubber tree fungal community (a). Significant differences between seasons in each sampling area are marked by stars. (b) Principal coordinates analysis of taxonomic similarity based on Bray-Curtis distances (OTU level). P, phyllosphere; PE, leaf endosphere; R, rhizosphere; RE, root endospher; RP, rhizoplane; S, soil. ***P < 0.001; **P < 0.01; and *P < 0.05.

In terms of alpha diversity, the observed OTU richness in the phyllosphere, rhizosphere, rhizoplane, and soil compartments were roughly equal, while the leaf endosphere showed significantly higher alpha diversity in Banna compared to Hainan, while the root endosphere exhibited the opposite trend (Fig. S5a; Table S2). When considering the seasonal effect, the observed OTU richness of fungal communities in the leaf endosphere (*P* < 0.01), root endosphere (*P* < 0.05), and rhizoplane (*P* < 0.01) were significantly higher during the rainy season compared to the dry season (Fig. S5b; Table S2). However, when analyzing the seasonal effect separately, distinct patterns were observed. In Banna, the richness of all compartments was significantly higher during the rainy season compared to the dry season (Fig. 3a). In Hainan, although the richness of the root endosphere and rhizoplane were similar between the rainy and dry seasons, and the leaf endosphere showed significantly higher richness during the rainy season, the phyllosphere, rhizosphere, and soil compartments exhibited significantly higher richness during the dry season (Fig. 3a). Neither Shannon nor Evenness index observed significant differences in Banna (Fig. S6). Only the Shannon diversity of leaf endosphere showed significantly higher, yet soil was significantly lower than the dry season in Hainan (Fig. S6a). Therefore, differences in Shannon and Evenness index between the rainy and dry soils were not considered in further analyses. Overall, the trend in richness was higher in the dry season compared to the rainy season in Hainan (Fig. 3a).

When analyzing the microbial composition based on the Bray-Curtis distance, we observed that geographical location had a significant effect on the beta diversity of fungal communities in all compartments (*P* < 0.01), while seasonal effects were not significant (Fig. 3). For instance, geographical location accounted for 6.62%, 7.35%, 9.13%, 8.07%, 17.67%, and 18.78% of the variation in the soil, rhizosphere, rhizoplane, root endosphere, phyllosphere, and leaf endosphere, respectively. Additionally, we observed a much greater effect of site variation compared to seasonal variation in all compartments (Fig. S7). In summary, our findings indicate that geographical location/site has a significant impact on fungal composition, while season does not.

### Drives of environmental factors in shaping rubber tree fungal community

**Fig 4:**
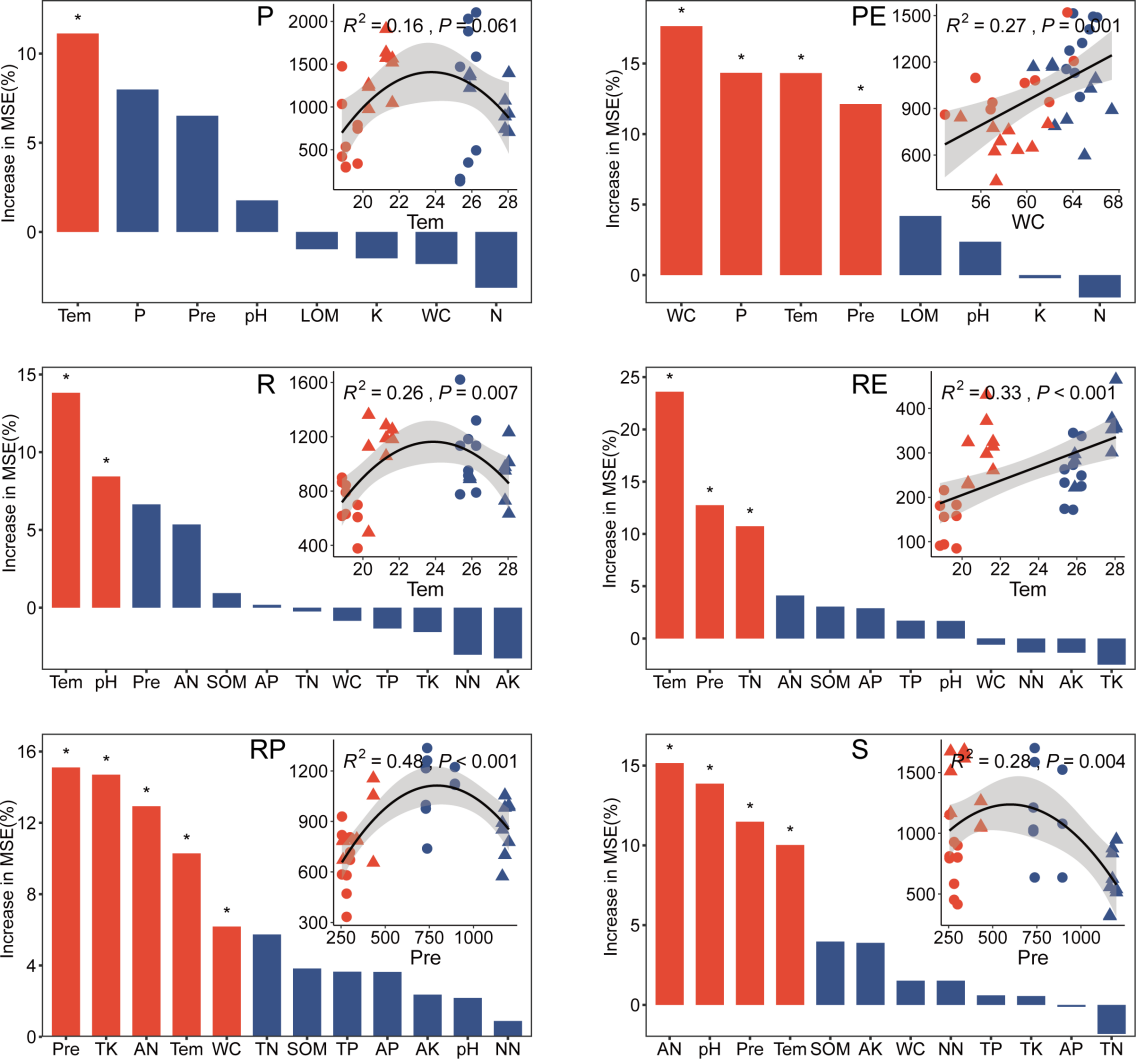
Drivers of soil bacterial alpha diversity across different compartments. Random forest (RF) analyses identifying the importance of potential predictors of fungal richness. RF Importance = Increase in % mean square error. Red and blue columns represent *P* < 0.05 and *P* > 0.05, respectively. Relationships between environmental factors and the bacterial alpha diversity was estimated via linear least-squares regression analysis. Solid circle (red: dry season; blue: rainy season) indicate samples from Banna; Solid triangle (red: dry season; blue: rainy season) indicate samples from Hainan. AK, available potassium; AN, Ammonium nitrogen; AP, available phosphorus; K, leaf potassium; LOM, leaf organic matter; N, leaf nitrogen; NN, nitrate nitrogen; P, leaf phosphorus; PE, leaf endosphere; pH, soil pH; Pre, precipitation; RE, root endosphere; R, rhizosphere; RP, rhizoplane; S, soil; SOM, soil organic matter; Tem, temperature; TK, total potassium; TN, total nitrogen; TP, total phosphorus; WC, water content.

Among all environmental factors, leaf WC, temperature, and precipitation were identified as the most important predictors of fungal alpha diversity in different compartments (Fig. 4; Fig. S5b). These findings were further supported by simple linear and nonlinear regression analyses. For instance, significant and positive simple linear regressions were observed between leaf WC, temperature, and fungal alpha diversity in the leaf endosphere and root endosphere compartments. Additionally, a similar mid-peak pattern was observed in four datasets, where richness peaked at mid-temperature in the phyllosphere and rhizosphere compartments, and peaked at mid-precipitation in the soil and rhizoplane compartments. These results indicate that climatic factors play a significant role in driving the alpha diversity of fungi in rubber tree ecosystems.

**Fig 5:**
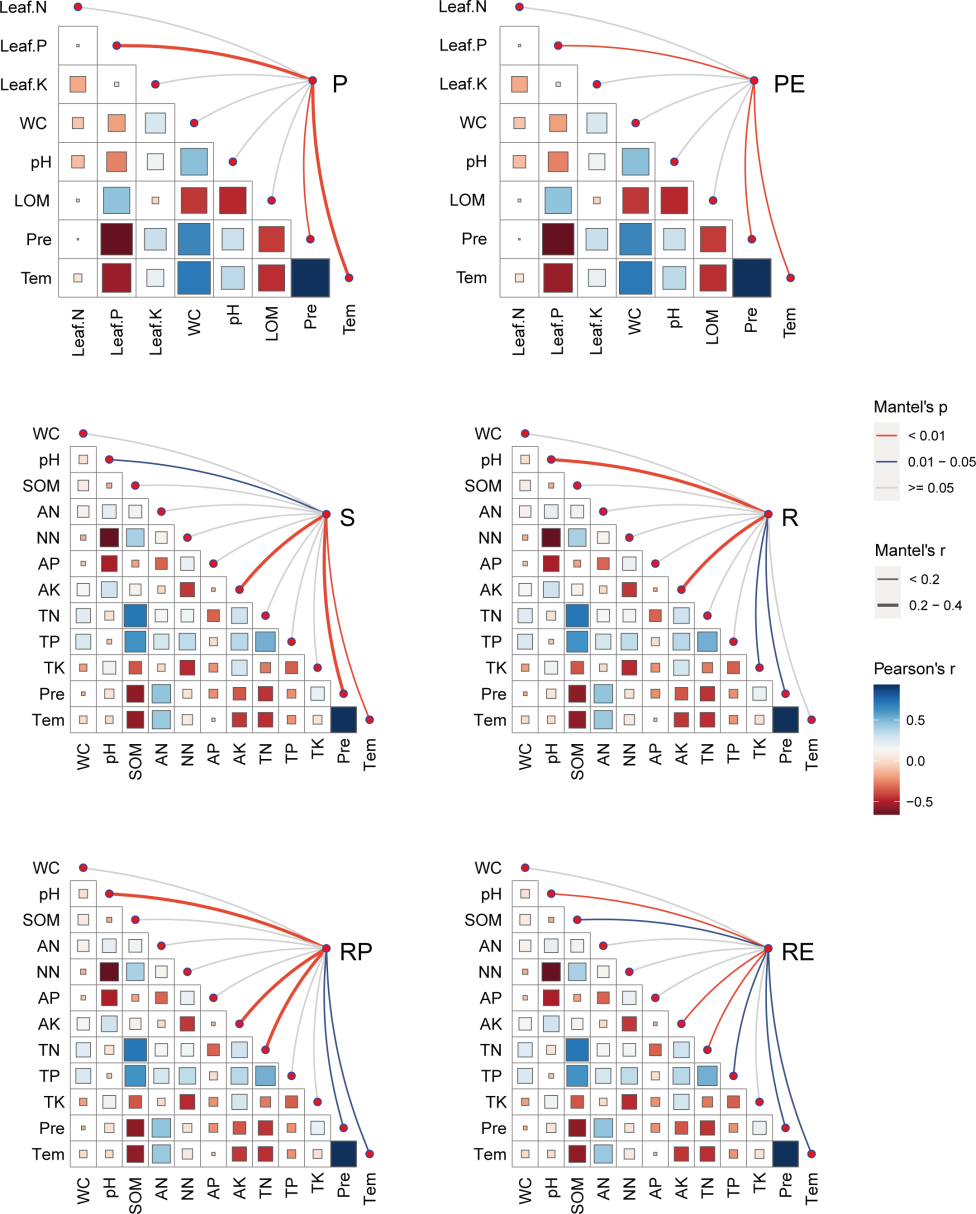
Correlation analysis among edaphic properties, climatic characteristics, and microbial communities in the rubber tree microbial community determined using the Mantel test. The color of the line represents the significance of the correlations (*P* values). The width of the line represents the size of the correlation coefficients (Mantel’s *r*). The size and color of the squares represent the values of the correlation coefficients (Pearson’s *r*). AK, available potassium; AN, ammonium nitrogen; AP, available phosphorus; leaf.K, leaf potassium; leaf.N, leaf nitrogen; leaf.P, leaf phosphorus; LOM, leaf organic matter; NN, nitrate nitrogen; pH, soil pH; pre, precipitation; SOM, soil organic matter; Tem, temperature; TK, total potassium; TN, total nitrogen; TP, total phosphorus; WC, water content.

**Fig 6:**
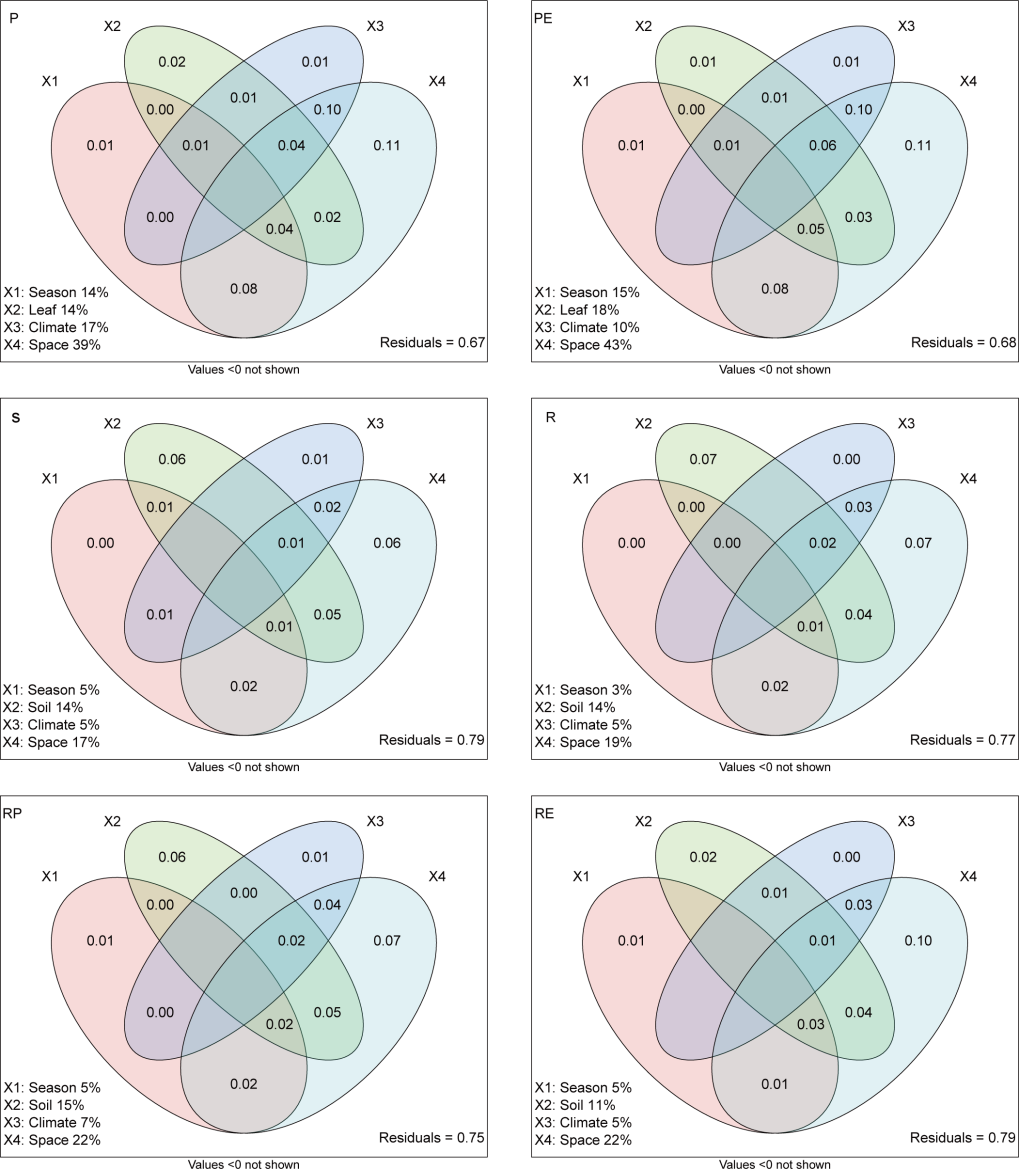
Variation partitioning analysis (VPA) showing the effects of leaf, soil properties, climatic factors, and geographic factors, and season, on the microbial community composition in the microbial beta diversity. The values indicate the percentage of variation significantly explained by each section.

The major physicochemical and climatic factors to the fungal composition were further identified using the Mantel tests. Of the most important environmental factors contributing to the leaf composition, leaf P had the largest observed effect, followed by temperature and precipitation, while soil AK affected the composition most in all root-associated compartments (Fig. 5; Table S3). Specifically, temperature and precipitation were significantly correlated with the fungal communities in different compartments to a certain extent (Table S3). The contributions of seasonal, environmental, climatic, and geographic variables to the variation in fungal composition were quantified by VPA. Geographic factors were better predictors of fungal composition than seasonal, environmental, and climatic ones (Fig. 6), confirming a stronger effect of spatial variation in driving the composition of soil fungi.

## DISCUSSION

Revealing the spatiotemporal pattern of microbial communities is a fundamental topic in ecology, which have been explored extensively in microbial ecology over the last two decades (38). Furthermore, the significant effect of the seasonal change in regulating the alpha diversity of soil bacteria and fungi was found at the regional scale (Hainan) in our previous study (6). Still, very few studies have mapped the temporal and spatial distribution changes of microbial communities of the soil–plant continuum. Here, we explored the spatiotemporal of soil–plant continuum fungal community in the rubber tree across different niches and regions, doing so can help to clarify the drivers of space and seasonal change upon microbial community variations. Our findings provide robust empirical evidence that the spatiotemporal variation of fungal diversity in rubber trees was mainly shaped by seasonal change, but only spatial impacts significantly altered microbial beta diversity.

Our results demonstrate that the alpha diversity of soil–plant continuum fungal community is highly dependent on seasonal changes, which is in line with previous studies that have observed significant seasonal variations in microbial diversity (20, 39–42). In our study, we provide evidence suggesting that climatic factors play a crucial role in mediating the seasonal variation of fungal alpha diversity. Climatic factors have been identified as the best predictors of soil fungal richness and community composition at a global scale (43, 44). Importantly, our study further extends this earlier observation at the soil–plant continuum and now provides widespread evidence that climatic factors mediate the alpha diversity of plant microbiome. Among the environmental variables, we found that temperature and precipitation were the dominant predictors for fungal richness, which has also been found in numerous previous studies (43–48). Notably, the alpha diversity is sensitive to temperature and precipitation, displaying an unimodal pattern in most compartments, except for the leaf endosphere and root endosphere which showed a linear pattern. Given that temperature and precipitation are generally higher during the rainy season and higher in Hainan compared to Banna, this further supports the observed unimodal pattern in most compartments. Thus, it is not surprising to find that the contrasted seasonal change pattern in Hainan and Banna. Furthermore, we observed a stronger seasonal effect compared to a geographical location effect on some environmental predictors. Specifically, leaf physicochemical factors such as WC and climatic factors such as temperature and precipitation were primarily influenced by seasonal changes, contributing to the mechanisms driving microbial seasonal variations.

In contrast, we provide an evidence that the beta diversity of soil–plant continuum only showed a geographical variation pattern. This aligns with previous studies that have shown spatial factors to be more important in shaping soil microbial communities across large spatial scales (6, 40, 49, 50). The effect of site location was far more influential than the seasonal change in regulating the communities of both soil bacteria and fungi at the regional scale (only in Hainan) in our previous study (6). Given that biogeographic patterns variation of community similarity indicates the influence of historical factors (51, 52), compared to our previous studies (research in Hainan soil samples) (6), the stronger geographical variation pattern suggests that the pronounced impact of historical event on fungal community structure in soil–plant continuum of rubber tree. For fungal beta diversity, P and soil AK were the most important factors in leaf and soil samples, respectively. Besides, major taxa belonging to Dothideomycetes and Eurotiomycetes in leaf samples, according to their life strategies, which have been assigned as copiotrophic fungi, this might explain why leaf samples responded to altered leaf nutrients (e.g., leaf P), which have already been demonstrated (53–56). It is worth noting that leaf P and AK which is a highly localized variable, were evidently stronger affected by sampling sites. In all, we found a stronger geographic location effect than seasonal effect upon fungal beta diversity estimates. Furthermore, geographic factors contributed a larger proportion of variation relative to edaphic and climatic factors to the beta diversity of rubber leaf than soils that of soil–plant continuum, indicating a stronger effect of stochastic processes in driving the beta diversity of rubber leaf. These results were also confirmed by the VPA models and Mantel test, and are also consistent with previous observations (57).

### Conclusion

Our results demonstrate that the alpha diversity is highly dependent on seasonal changes, while beta diversity only showed a geographical variation pattern. Especially, our work showed that spatiotemporal variation in fungal community alpha and beta diversity was mainly driven by climatic factors (temperature and precipitation) and soil properties (e.g., AK), leaf properties (e.g., P), respectively. Moreover, the leaf P and AK were mainly explained by the geographic location effect rather than the seasonal effect, and climatic factors showed opposite pattern. Taken together, our study provides empirical evidence for the distinct spatiotemporal patterns and driving factors between alpha and beta diversity in the fungal community of the soil–plant continuum in rubber trees.

## Data Availability

Raw sequencing data for fungal communities have been submitted to the NCBI Sequence Read Archive (SRA) database (accession number: SRP342019).

## SUPPLEMENTAL MATERIAL

Supplemental material spectrum.02097-24-s0001.pdf Supplemental methods; Fig. S1 to S7; Tables S2 and S3.

Table S1 spectrum.02097-24-s0002.csv The fungal OTU table.

ASM does not own the copyrights to Supplemental Material that may be linked to, or accessed through, an article. The authors have granted ASM a non-exclusive, world-wide license to publish the Supplemental Material files. Please contact the corresponding author directly for reuse.
