# Supplementary material for: Distinct spatiotemporal patterns between fungal alpha and beta diversity of soil–plant continuum in rubber tree
Source: Microbiol Spectr. 2024 Dec 27;13(2):e02097-24. doi: 10.1128/spectrum.02097-24 (PMC11792516; doi:10.1128/spectrum.02097-24)
Supplement: Supplemental material — Supplemental methods; Fig. S1 to S7; Tables S2 and S3. [file spectrum.02097-24-s0001.pdf]

Distinct spatiotemporal patterns between fungal alpha and beta diversity of soil–plant continuum in rubber tree

Wei Yaqing[a,b],Lan Guoyu[a,b*], Wu Zhixiang[a,b]

The following supporting information is available for this article:

Method S1

Sampling

In each sampling plot, three rubber trees, with a distance about 100 meters from each other, were selected as our study objectives. Top soil (0-20 cm) samples were collected from at the middle of two rubber trees and the center of three rubber trees (Figure S1B). After sieving, they were mixed to form a composite soil sample. Root samples were selected from the four directions (i.e., north, south, east, and west) of each rubber tree, then mixed to form a single composite sample (Figure S1B). Rhizosphere soil was manually separated from the roots by shaking, while leaving rhizoplane soil (~1 mm thick layer of soil) still attached to the roots (1-3). Leaves were collected in the same time from branches 2 m away from the center of the trunk and 12 m above the ground (Figure S1B). Leaves were selected from each of the four cardinal directions and all sampled leaves from the three trees were mixed for a single composite sample.

A total of 36 leaf samples, 36 soil samples and 36 root samples were collected in the two seasons (rainy season and dry season), and all samples were transported to the laboratory on ice and placed in sterile PBS solution to isolate epiphytic and endophytic microbes within 24h.

To collect the rhizoplane soil sample, place 5 g of roots root in a 50 ml sterile centrifuge tube containing 35 mL of sterile PBS and subjected to sonication using a sonication bath for 1 min, then shaken at 200 rpm for 4 min, this process is repeated 2-3 times. The buffer with microbial cells is then passed through a 0.22 μm filter membrane, and the resulting filter membrane was stored in a sterile centrifuge tube at −80 ℃ in preparation for microbial sequencing analyses. To collect the phyllosphere

sample, place 15 g of leaves in a 250 ml sterile glass vial containing 200 mL of sterile PBS and subjected to sonication using a sonication bath for 1 min, then shaken at 200 rpm for 4 min, this process is repeated 2-3 times. The buffer with microbial cells is then passed through a 0.22 μm filter membrane, and the resulting filter membrane was stored in a sterile centrifuge tube at −80°C in preparation for microbial sequencing analyses.

For leaf and root endophytic sample, most of the microbial cells on the plant surface were removed by treatment as described above, and the leaves or roots obtained were washed with sterile water for the next step of surface disinfection. The main steps are: soak in 70% alcohol for 5 minutes, then in 5.25% sodium hypochlorite solution for 5 minutes, then in 70% alcohol for 30 seconds, and finally wash with sterile water at least 5 times to avoid alcohol residue. The leaves and roots samples were stored at −80 ℃ in preparation for microbial sequencing analyses.

Therefore, we obtained 216 samples, including those from the phyllosphere (36 samples), leaf endosphere (36 samples), soil (36 samples), rhizosphere (36 samples), rhizoplane (36 samples), and root endosphere (36 samples) compartments.

For leaf sample, TN was determined using micro-Kjeldahl digestion followed by steam distillation. TP and TK were assessed using NaOH digestion. WC content and LOM content were assessed using the gravimetric method. Leaf pH was measured in a leaf/water suspension (1:2.5, w/w) using a pH meter. Soil pH was measured in a 1:1 soil: water mixture. Soil moisture was measured gravimetrically. Soil TN was determined using a micro-Kjeldahl digestion followed by steam distillation. Total phosphorus (TP) and total potassium (TK) were digested with NaOH. Nitrate nitrogen (NN) and ammonium nitrogen (AN) were determined by steam distillation and indophenol-blue colorimetry, respectively. Soil samples were extracted with NaHCO3, and the extracts then used to measure the available soil phosphorus (AP) using molybdate-blue colorimetry. For soil potassium (AK), soil samples were first extracted with ammonium acetate, before loading the extracts onto an atomic absorption spectrometer with ascorbic acid as a reductant (4)

Supplementary figures

Figure S1 Study sites on Hainan Island and Xishuangbanna. Red solid circles indicate study sites on the maps. Each site was further divided into 3 plots, totaling 18 sampling plots.

Figure S2 Spatiotemporal changes in fungal community composition (class level) in different compartments of rubber trees.

Figure S3 Environmental variables in dry and rainy seasons between two locations of rubber tree leaves. The significant differences between seasons and locations were detected by two-way ANOVA. N: Total nitrogen, P: Total phosphorus, K: Total potassium, WC: Water content, LOM: Leaf organic matter.

Figure S4 Environmental variables in dry and rainy seasons among the six sampling sites of rubber tree leaves. The significant differences between seasons and locations were detected by two-way ANOVA. WC: Water content, SOM: Soil organic matter, pH: Soil pH, AN: Ammonium nitrogen, NN: Nitrate nitrogen AP: Available phosphorus, AK: Available potassium, TN: Total nitrogen, TP: Total phosphorus, TK: Total potassium.

Figure S5 Boxplots showing the number of OTUs between the two locations (a) and seasons (b). Leaf.WC: Leaf water content.

Figure S6 Shannon (a) and evenness (b) diversity of rubber tree fungal community.

Figure S7 Boxplots showing the number of OTUs between the two locations (a) and seasons (b).

Figure S7 Principal coordinates analysis (PCoA) of taxonomic similarity based on Bray-Curtis distances (OTU level). Six compartments in Hainan (a) and Banna (b). LD: Ledong, DZ: Danzhou, WN: Wanning; MP: Mengpeng, JH: Jinghong, ML: Menglun.

Supplementary tables

Table S1 The OTUs table.

Table S2 The significant effect of location and season on fungal alpha diversity was detected by two-way ANOVA.

Table S3 Mantel test results for the correlation between community similarity and environmental.

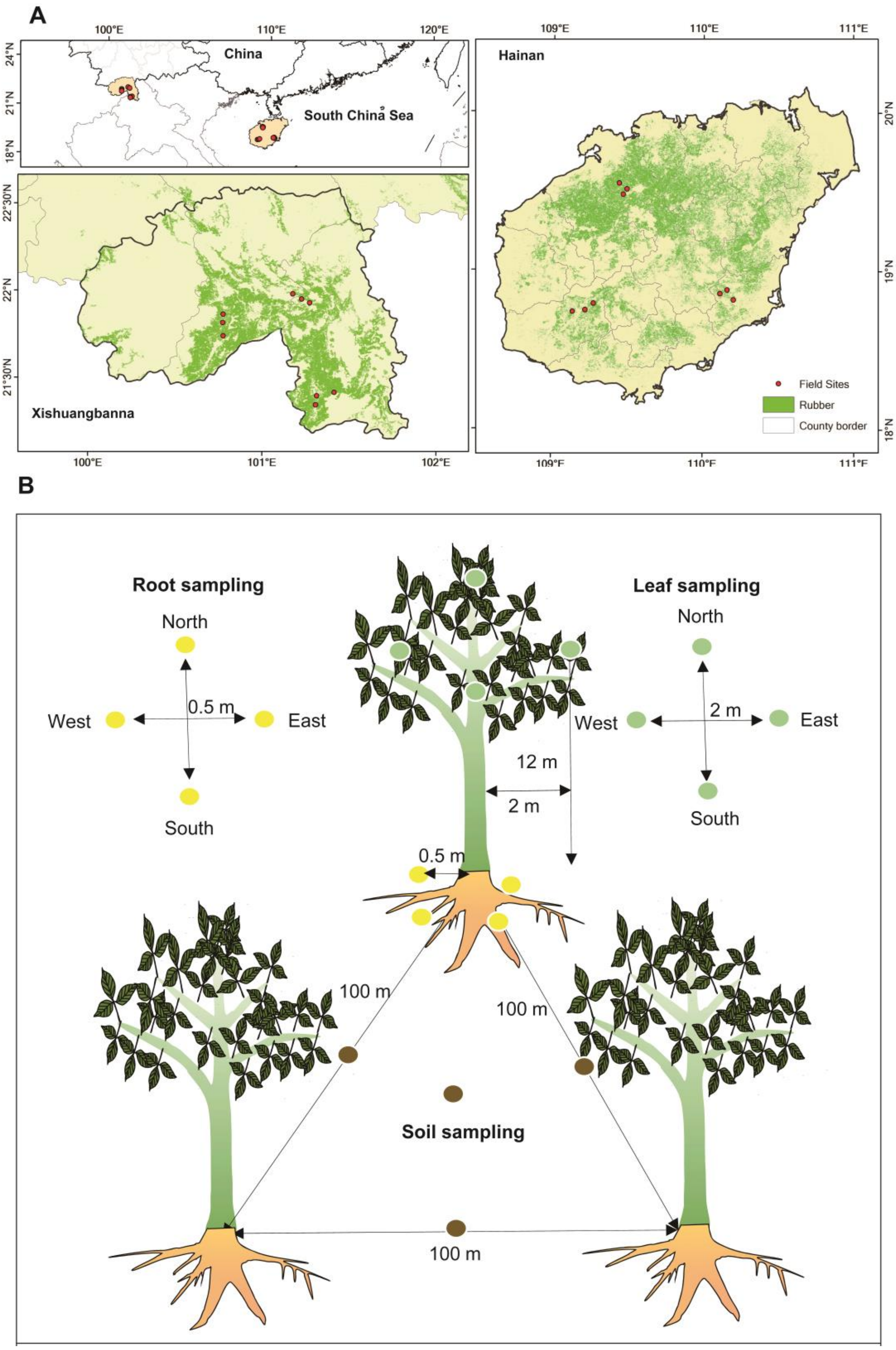

Figure S1 Study sites (A) and sampling design (B) on Hainan Island and in Xishuangbanna. Red solid circles indicate study sites on the maps. Each site was further divided into 3 plots, totaling 18 sampling plots. For each plot, we selected three trees and sampled root (yellow solid circles) and leaves (green solid circles) from all four cardinal directions. The brown solid circles indicate sampling locations of soil.

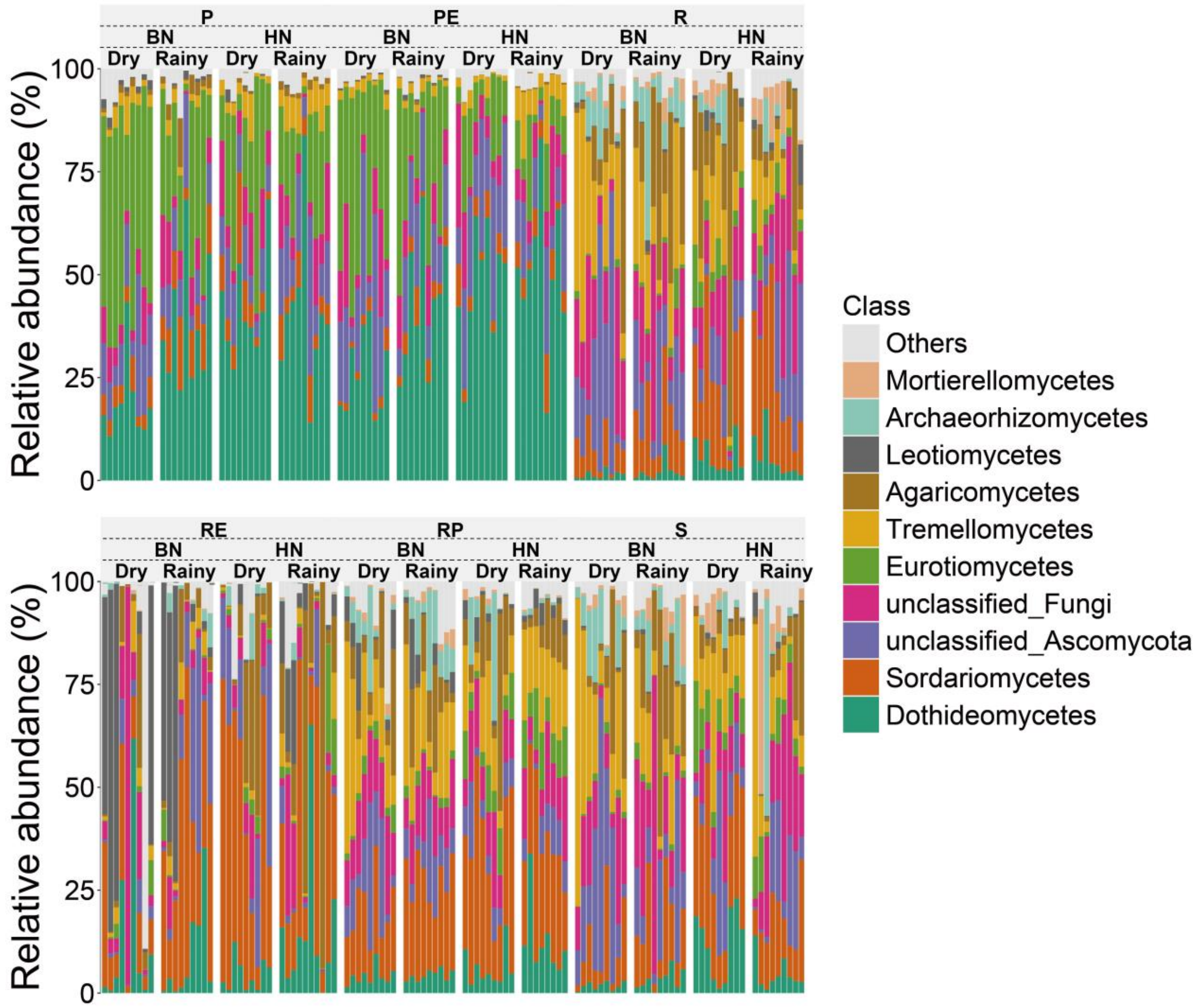

Figure S2 Spatiotemporal changes in fungal community composition (class level) in different compartments of rubber trees.

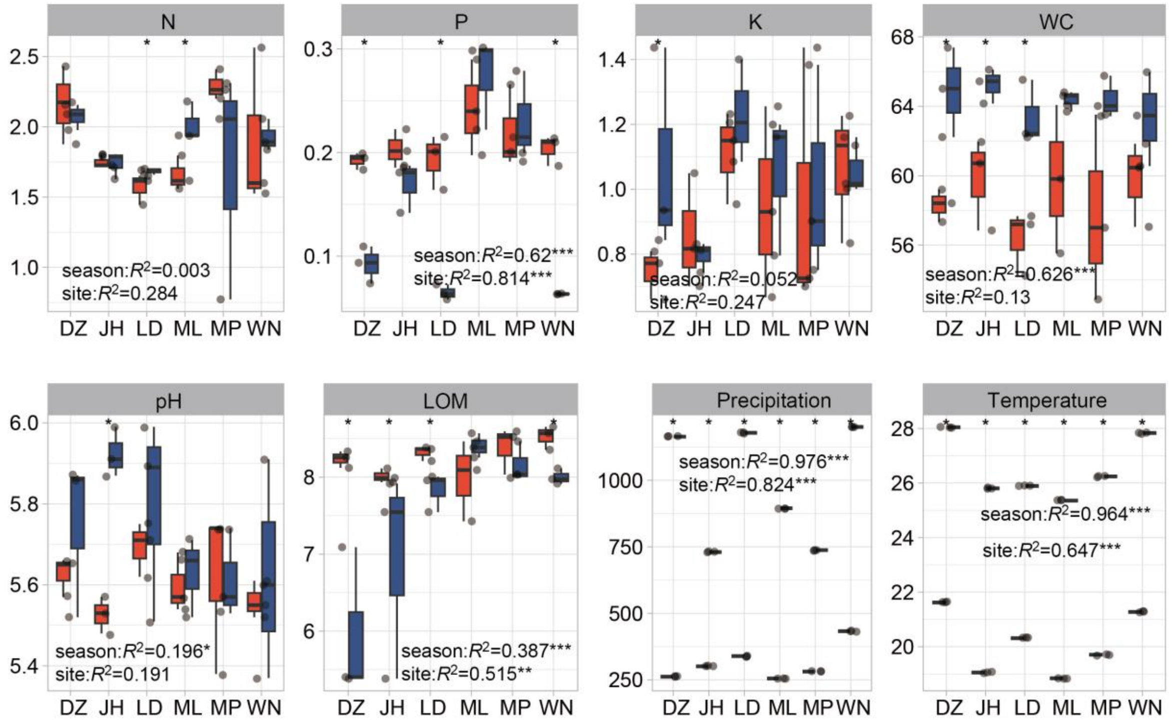

Figure S3 Environmental variables in dry and rainy seasons among the six sampling sites of rubber tree leaves. The significant differences between seasons and locations were detected by two-way ANOVA. N: Total nitrogen, P: Total phosphorus, K: Total potassium, WC: Water content, LOM: Leaf organic matter. *, $P < 0.05$; **, $P < 0.01$, ***, $P < 0.001$

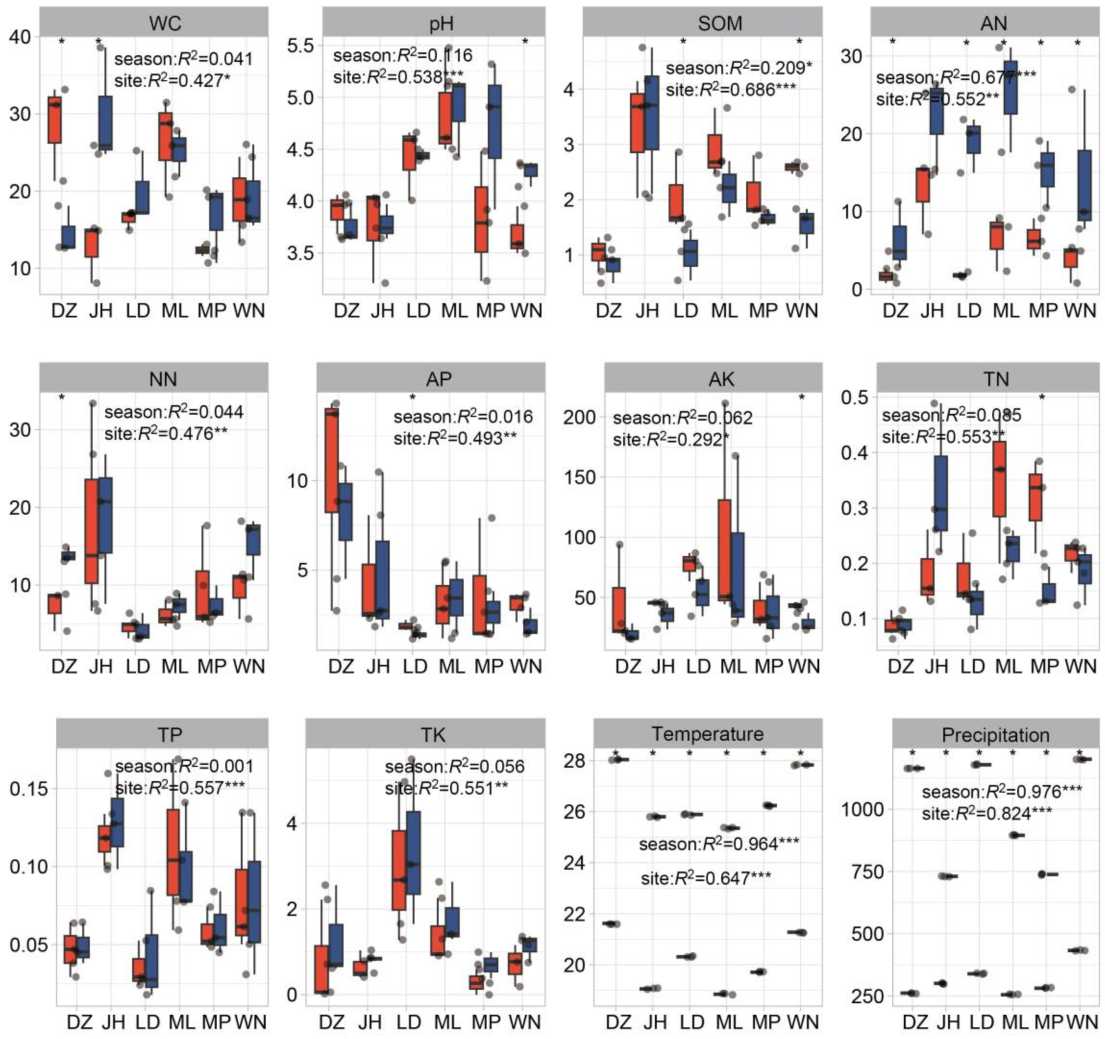

Figure S4 Environmental variables in dry and rainy seasons among the six sampling sites of rubber tree soils. The significant differences between seasons and locations were detected by two-way ANOVA. WC: Water content, SOM: Soil organic matter, pH: Soil pH, AN: Ammonium nitrogen, NN: Nitrate nitrogen AP: Available phosphorus, AK: Available potassium, TN: Total nitrogen, TP: Total phosphorus, TK: Total potassium. *, $P < 0.05$; **, $P < 0.01$, ***, $P < 0.001$

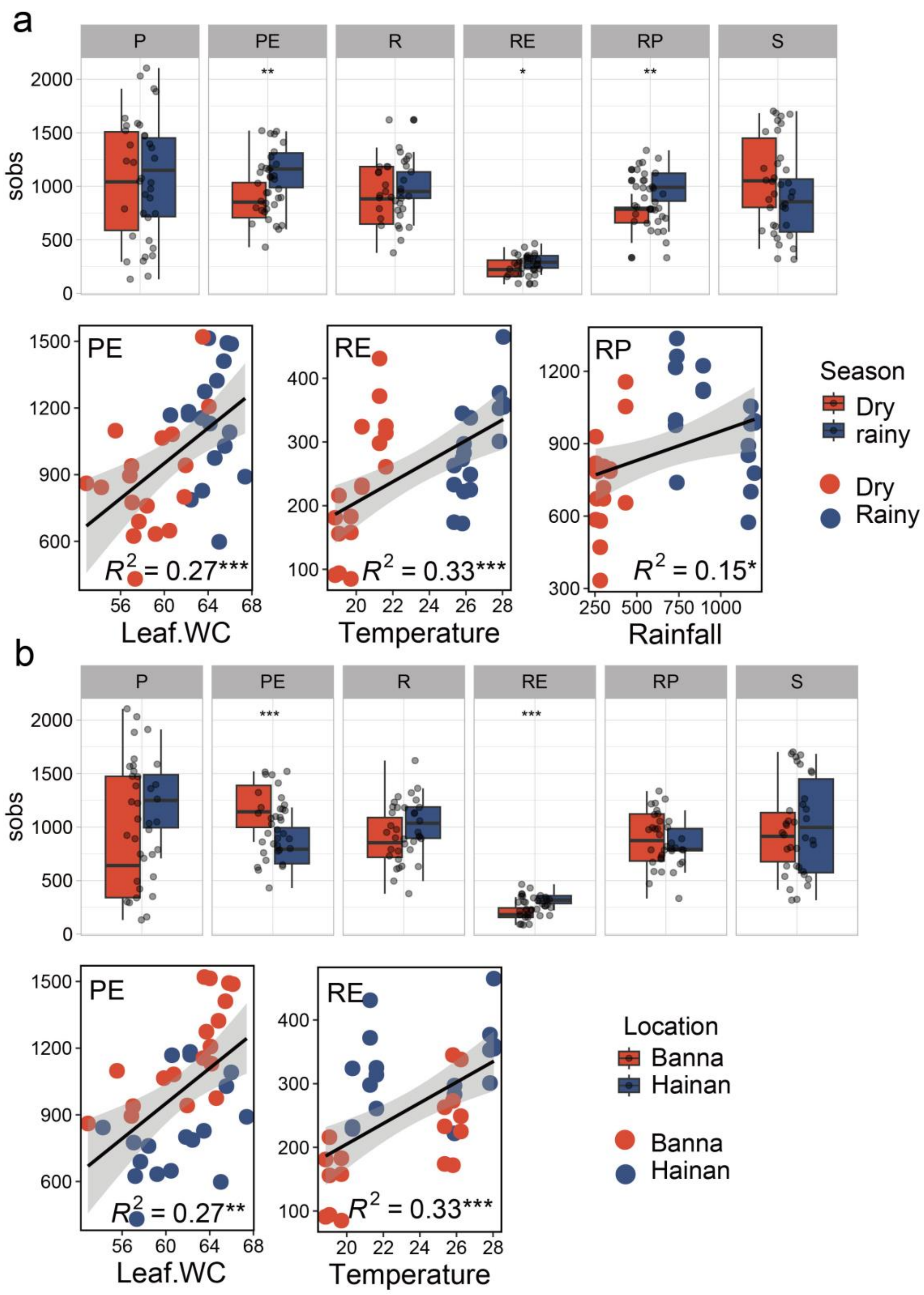

Figure S5 Boxplots showing the number of OTUs between the two locations (a) and seasons (b). ***P < 0.001; **P < 0.01; *P < 0.05. Leaf.WC: Leaf water content.

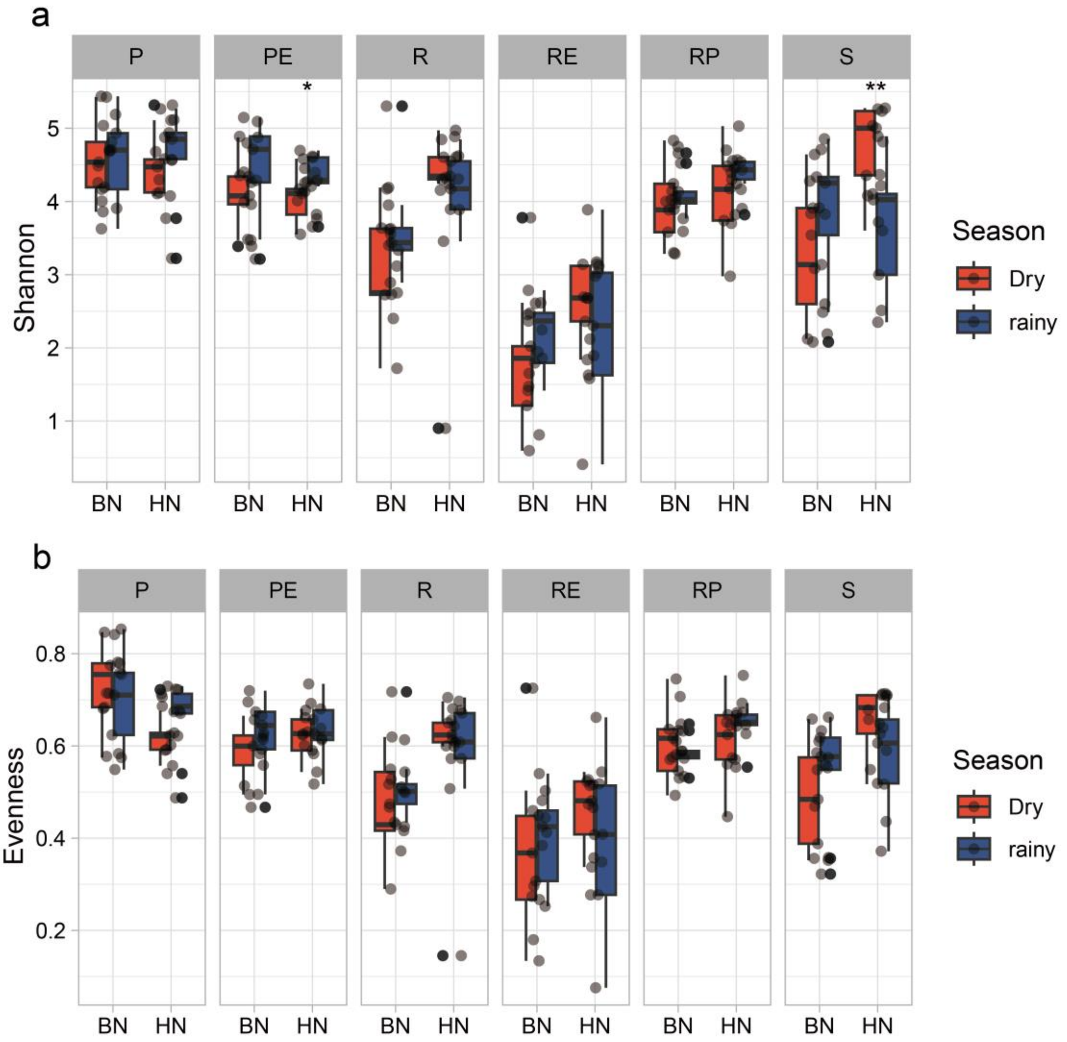

Figure S6 Shannon (a) and evenness (b) diversity of rubber tree fungal community. Significant differences between seasons in each sampling area are marked by stars. $^{**}P < 0.01$; $^{*}P < 0.05$.

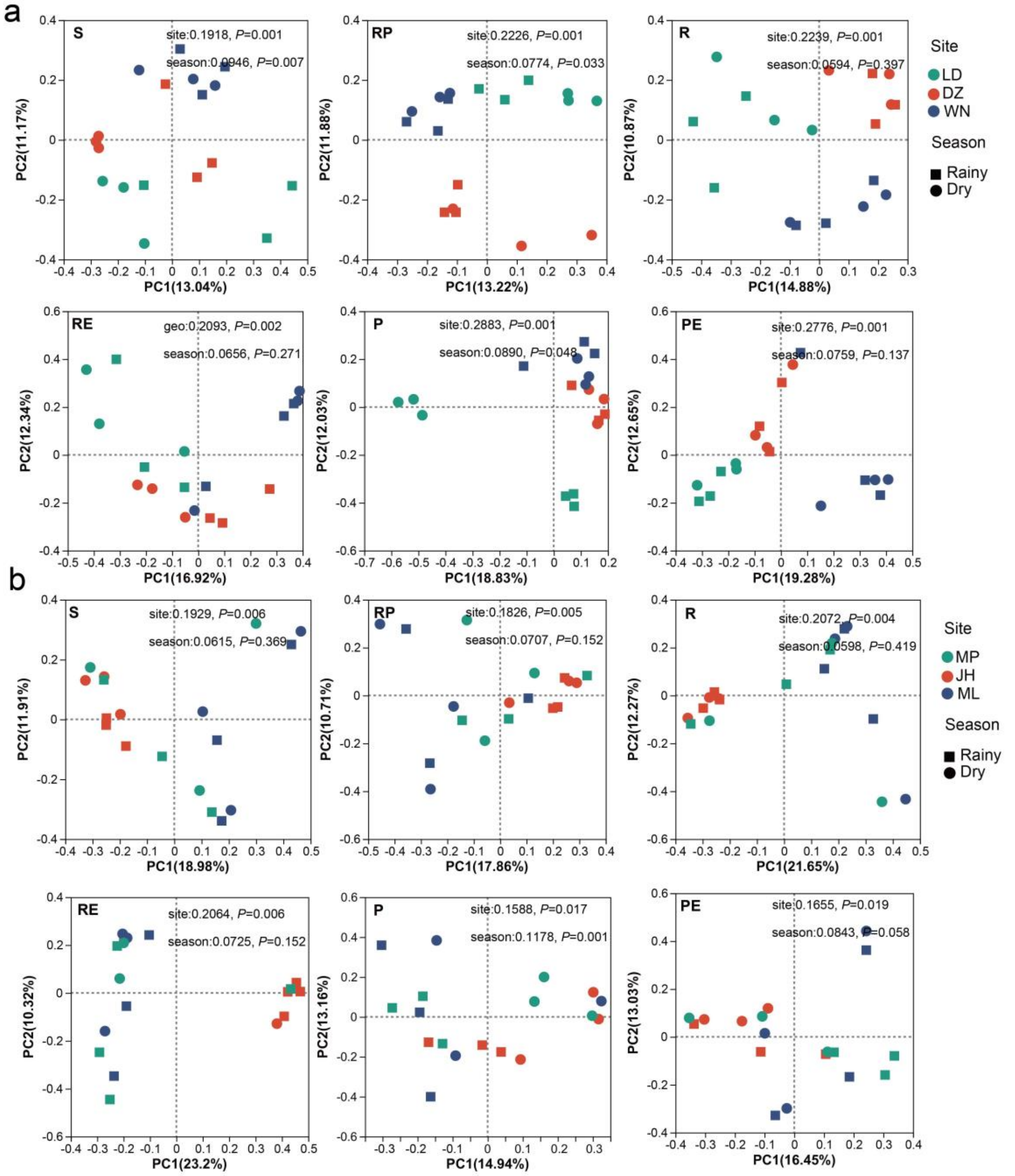

Figure S7 Principal coordinates analysis (PCoA) of taxonomic similarity based on Bray-Curtis distances (OTU level). Six compartments in Hainan (a) and Banna (b). LD: Ledong, DZ: Danzhou, WN: Wanning; MP: Mengpeng, JH: Jinghong, ML: Menglun. ***P < 0.001; **P < 0.01; *P < 0.05

Table S2 The significant effect of location and season on fungal alpha diversity was detected by two-way ANOVA.

		Df	Sum Sq	H	Pr(>F)
Phyllosphere					
	Location	1	373.78	3.3674	0.066
	Season	1	4.00	0.0360	0.849
	**Location: Season**	**1**	**625.00**	**5.6306**	**0.017**
	Residuals	32	2882.22		
Leaf endosphere					
	**Location**	**1**	**1573.4**	**14.1752**	**0.00017**
	**Season**	**1**	**841.0**	**7.5766**	**0.00591**
	Location: Season	1	25.0	0.2252	0.63509
	Residuals	32	1445.6		
Soil					
	Location	1	9.00	0.0811	0.77582
	Season	1	205.44	1.8511	0.17366
	**Location: Season**	**1**	**2146.78**	**19.3428**	**0.00001**
	Residuals	32	1523.28		
Rhizosphere					
	Location	1	342.25	3.0841	0.07906
	Season	1	75.11	0.6769	0.41067
	**Location: Season**	**1**	**1284.03**	**11.5708**	**0.00067**
	Residuals	32	2182.61		
Rrhizoplane					
	Location	1	26.69	0.2405	0.62383
	**Season**	**1**	**1024.00**	**9.2264**	**0.00239**
	**Location: Season**	**1**	**633.36**	**5.7067**	**0.01690**
	Residuals	32	2200.44		
Root endosphere					
	**Location**	**1**	**1736.11**	**15.6427**	**0.000077**
	**Season**	**1**	**484.00**	**4.3609**	**0.036772**
	Location: Season	1	177.78	1.6018	0.205647
	Residuals	32	1486.61		

Table S3 Mantel test results for the correlation between community similarity and environmental. WC: Water content, LOM: Leaf organic matter, SOM: Soil organic matter, pH: Soil pH, AN: Ammonium nitrogen, NN: Nitrate nitrogen AP: Available phosphorus, AK: Available potassium, TN: Total nitrogen, TP: Total phosphorus, TK: Total potassium.

Compartment	Factor	r	p
Phyllosphere	**Leaf.P**	**0.254**	**0.001**
	**Temperature**	**0.200**	**0.001**
	**Rainfall**	**0.190**	**0.001**
	Leaf.K	0.045	0.193
	WC	0.041	0.217
	Leaf.N	-0.039	0.717
	pH	-0.044	0.756
	LOM	-0.125	0.955
Leaf endosphere	**Leaf.P**	**0.167**	**0.001**
	**Rainfall**	**0.140**	**0.009**
	**Temperature**	**0.126**	**0.008**
	WC	0.053	0.148
	Leaf.K	0.051	0.135
	pH	-0.027	0.667
	LOM	-0.066	0.81
	Leaf.N	-0.101	0.956
Soil	**AK**	**0.312**	**0.005**
	**Rainfall**	**0.225**	**0.001**
	**pH**	**0.195**	**0.019**
	TN	0.127	0.12
	**Temperature**	**0.121**	**0.008**
	TK	0.108	0.162
	AN	0.106	0.092
	TP	0.051	0.273
	AP	0.015	0.419
	SOM	0.004	0.461
	WC	-0.078	0.823
	NN	-0.103	0.83
Rhizosphere	**AK**	**0.313**	**0.001**
	**pH**	**0.261**	**0.003**
	**TK**	**0.191**	**0.043**
	TN	0.125	0.109
	**Rainfall**	**0.105**	**0.032**
	TP	0.090	0.148
	AN	0.067	0.179
	Temperature	0.022	0.301

	SOM	-0.011	0.526
	NN	-0.019	0.531
	AP	-0.043	0.631
	WC	-0.079	0.824
Rhizoplane	**AK**	**0.329**	**0.002**
	**pH**	**0.293**	**0.001**
	**TN**	**0.271**	**0.005**
	WC	0.109	0.079
	**Rainfall**	**0.108**	**0.034**
	**Temperature**	**0.100**	**0.023**
	TK	0.093	0.189
	TP	0.091	0.13
	SOM	0.076	0.19
	AP	-0.011	0.519
	NN	-0.060	0.704
	AN	-0.060	0.797
Root endosphere	**AK**	**0.190**	**0.001**
	**TN**	**0.177**	**0.001**
	**pH**	**0.154**	**0.003**
	**TP**	**0.128**	**0.015**
	**SOM**	**0.099**	**0.035**
	**Rainfall**	**0.091**	**0.029**
	**Temperature**	**0.085**	**0.035**
	WC	0.066	0.099
	AN	0.045	0.195
	TK	0.021	0.349
	AP	0.010	0.432
	NN	-0.008	0.531

References

1. Attia S, Russel J, Mortensen MS, Madsen JS, Sørensen SJ. 2022. Unexpected diversity among small-scale sample replicates of defined plant root compartments. Isme j 16:997-1003.
2. Richter-Heitmann T, Eickhorst T, Knauth S, Friedrich MW, Schmidt H. 2016. Evaluation of Strategies to Separate Root-Associated Microbial Communities: A Crucial Choice in Rhizobiome Research. Front Microbio 7.
3. Edwards J, Johnson C, Santos-Medellín C, Lurie E, Podishetty NK, Bhatnagar S, Eisen JA, Sundaresan V. 2015. Structure, variation, and assembly of the root-associated microbiomes of rice. Proc NatlAcad Sci USA 112:E911-20.
4. Chen J, Xu H, He D, Li Y, Luo T, Yang H, Lin M. 2019. Historical logging alters soil fungal community composition and network in a tropical rainforest. Forest Ecol Manag 433:228-239
